# Visibility of Intracranial Perforating Arteries Using Ultra-High-Resolution Photon-Counting Detector Computed Tomography (CT) Angiography

**DOI:** 10.3390/tomography10120136

**Published:** 2024-11-21

**Authors:** Takashi Okazaki, Tetsu Niwa, Ryoichi Yoshida, Takatoshi Sorimachi, Jun Hashimoto

**Affiliations:** 1 Department of Diagnostic Radiology, Tokai University School of Medicine, 143 Shimokasuya, Isehara 259-1193, Japan; okazakit@tokai.ac.jp (T.O.); junhashi@tokai.ac.jp (J.H.); 2 Department of Radiology, Tokai University Hospital, 143 Shimokasuya, Isehara 259-1193, Japan; r.yoshida@tokai.ac.jp; 3 Department of Neurosurgery, Tokai University School of Medicine, 143 Shimokasuya, Isehara 259-1193, Japan; sorimachi@tokai.ac.jp

**Keywords:** photon-counting detector CT, intracranial perforating arteries, CT angiography, ultra-high-resolution CT

## Abstract

**Background/Objectives:** Photon-counting detector computed tomography (PCD-CT) offers energy-resolved CT data with enhanced resolution, reduced electronic noise, and improved tissue contrast. This study aimed to evaluate the visibility of intracranial perforating arteries on ultra-high-resolution (UHR) CT angiography (CTA) on PCD-CT. **Methods:** A retrospective analysis of intracranial UHR PCD-CTA was performed for 30 patients. The image quality from four UHR PCD-CTA reconstruction methods [kernel Hv40 and Hv72, with and without quantum iterative reconstruction (QIR)] was assessed for the lenticulostriate arteries (LSAs) and pontine arteries (PAs). A subjective evaluation included peripheral visibility, vessel sharpness, and image noise, while objective analysis focused on the signal-to-noise ratio (SNR) and contrast-to-noise ratio (CNR). **Results:** Peripheral LSAs were well visualized across all reconstruction methods, with no significant differences between them. Vessel sharpness and image noise varied significantly (*p* < 0.0001); sharper LSAs and more noise were seen with kernel Hv72 compared to kernel Hv40 (*p* < 0.05). A similar pattern was observed for PAs, though peripheral visibility was lower than that for LSAs. The SNR and CNR were the highest in the presence of kernel Hv72 with QIR, and lowest with kernel Hv72 without QIR, compared to kernel Hv40 (*p* < 0.05). **Conclusions:** UHR PCD-CTA provided a good visualization of the intracranial perforating arteries, particularly LSAs. The vessel sharpness and image noise varied by reconstruction method, in which kernel Hv72 with QIR offered the optimal visualization.

## 1. Introduction

Computed tomography (CT) is one of the most widely used imaging modalities for whole-body examinations. Its applications for intracranial lesions include detecting acute infarctions, hemorrhages, tumors, and vascular abnormalities. CT angiography (CTA) is valuable for identifying vascular issues like stenosis, occlusion, aneurysms, hemorrhagic points, and vascular malformations [1,2,3,4]. However, conventional CTA provides limited vascular information in intracranial lesions due to the difficulty in visualizing small vessels like perforators.

Photon-counting detector (PCD)-CT is a newer technology that uses a direct X-ray conversion detector, where X-ray photon energies are directly converted into electronical signals [5,6,7,8,9,10,11]. PCD-CT reduces electronic noise and artifacts by using energy thresholds [8,12]. In addition, the PCD does not require reflective septa, unlike energy-integrated detectors, resulting in enhanced spatial resolution [13,14,15]. This technology allows for ultra-high-resolution (UHR) CT, with detector pixels measuring 0.151 × 0.176 mm^2^ at the isocenter [6,16], enabling 0.2 mm slice thickness reconstruction in CTA for detailed diagnostics [17,18]. We hypothesized that UHR PCD-CTA could be used to visualize small vessels, such as the lenticulostriate arteries (LSAs) and pontine arteries (PAs).

The LSAs typically branch from the middle cerebral artery (MCA) and supply the lateral portion of the caudate nucleus head, the entire putamen, the anterior limb, genu, and upper part of the internal capsule, as well as part of the corona radiata. The intermediate branches supply the anterior region of the LSA territory, while the lateral branches supply the posterior region. Perforators from the anterior cerebral artery, mainly Heubner’s artery, supply the inferomedial caudate head, the anteromedial putamen, the anterior lateral globus pallidus, and the anterior internal capsule. These regions correspond to the anterior and ventral basal ganglia. The PAs typically originate from the basilar artery and are classified based on vascular distribution, number of terminal branches, branching pattern, and segment origin from the basilar artery [19]. PAs are generally grouped into three types—paramedian, short circumferential, and long circumferential branches.

The LSAs and PAs are the key perforators involved in vascular conditions such as cerebral infarction, hemorrhage, and vascular malformations. Therefore, analyzing these perforators can aid in both pathophysiological and anatomical research. In addition, understanding their pathways is valuable, especially before surgical or endovascular procedures [20]. Visualizing thin perforator branches on CT allows for a more detailed evaluation of vascular disorders and related lesions. This study aimed to assess the visibility of intracranial perforating arteries using UHR PCD-CTA, focusing on the effects of different kernels and iterative reconstruction techniques.

## 2. Materials and Methods

### 2.1. Patients

A search was conducted in the radiology reporting system for patients who underwent CTA on PCD-CT between July 2023 and January 2024. The exclusion criteria were as follows: (1) severe artifacts caused by metals such as coils or postoperative devices (for partial artifacts, only non-affected areas were analyzed) and (2) large tumors interfering with the assessment region.

### 2.2. Ethical Approval

This study received approval from the Institutional Review Board for Clinical Research at Tokai University, with a waiver for informed consent (IRB No. 23R261).

### 2.3. CT Imaging

UHR CTA was performed using a dual-source PCD-CT (NAEOTOM Alpha; Siemens Healthcare, Forchheim, Germany). The scan parameters were as follows: a tube voltage of 120 kV, a collimation of 120 × 0.2 mm, a pitch of 0.85, a gantry rotation time of 0.5 s, and a CARE keV image quality level set to 230.

Following a pre-contrast CT, contrast-enhanced CT was conducted with Omnipaque 350 (GE Healthcare, Tokyo, Japan), which was administered through a 20-gauge needle inserted into the antecubital vein at a rate of 25.0/mgI/kg/s, along with a 20 mL saline chaser via a power injector (Dual Shot GX7; Nemoto Kyorindo, Tokyo, Japan). Bolus tracking was used, and the contrast-enhanced CTA scan began 6 s after detecting contrast enhancement in the internal carotid artery at the base of the posterior fossa.

**Figure 1:**
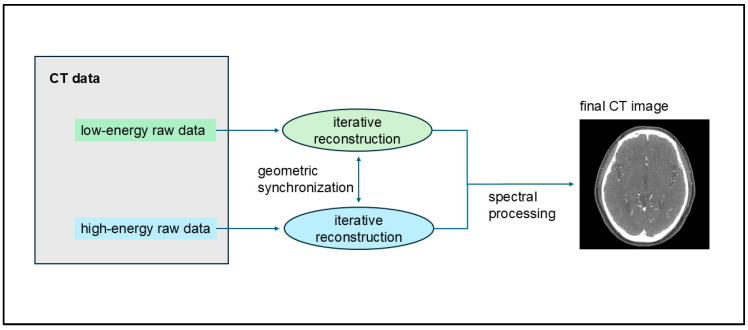
Process of quantum iterative reconstruction (QIR). QIR divides raw detector data into two energy-level streams, each undergoing its iterative loop for artifact cancelation and noise reduction. Synchronization points ensure precise geometric alignment between these streams, which undergo spectral processing to create spectral maps and monoenergetic images.

UHR CTA images were reconstructed using the Hv40 and Hv72 kernels, with and without quantum iterative reconstruction (QIR). QIR is a new model-based IR method designed specifically for PCD-CT [21,22,23,24,25,26]. In the QIR process, the raw detector data are divided into two streams based on energy levels, with each stream entering its iterative loop for artifact cancelation and noise reduction. To maintain precise geometric alignment between these energy levels, synchronization points are introduced in the projection and image data loops. Subsequently, these fully synchronized streams proceed through spectral processing to produce spectral maps and monoenergetic images [27] (Figure 1). QIR is installed in NAEOTOM Alpha; thus, selecting whether to use QIR and the strength of its application at image reconstruction is possible. QIR offers four levels of IR reconstruction strength. Herein, strength level 3 was used for QIR. The matrix size was 1024 × 1024 pixels, and the field of view was 200 × 200 mm^2^. All images were transferred to a workstation (*syngo*.via, 8.04, Siemens Healthcare, Forchheim, Germany).

### 2.4. Subjective Analysis

The quality of four types of UHR CTA images (using kernel Hv40 and Hv72, with and without QIR) was assessed on the workstation. Two board-certified radiologists (T.O. and T.N., with 12 and 25 years of neuroradiology experience, respectively) independently assessed the images. The radiologists were blinded to the kernel parameters and QIR usage and rated the visibility of the LSAs and PAs. CT images were presented randomly, and the observers rated the peripheral visibility, sharpness of the perforators, and image noise. The peripheral visibility of the perforators was rated as follows: 3—excellent, with the distal part visible near the cerebral ventricle (lateral ventricles for LSA and fourth ventricle for PA); 2—good, with more than half of the perforator visible from the branch to the ventricle; 1—poor, with less than half of the perforator visible; and 0—not visible. For multiple identified perforators, the most visible one was selected for rating. Vessel sharpness was rated as follows: 2—sharp; 1—slightly blurred; and 0—significantly blurred. Image noise was assessed as follows: 2—good, sufficient for diagnosis; 1—slight noise, but still diagnosable; and 0—excessive noise, not suitable for diagnosis. Images were displayed using a partial maximum intensity projection with 5 mm thickness. The radiologists assessed the images from various cross-sectional directions on the workstation, initially using a window level/width of 700/80 for vascular imaging, but they could adjust these settings as needed.

The number of LSAs branching from the MCA was counted separately for the right and left sides by two readers (T.O. and T.N.) working together to reach a consensus. Perforators smaller than 5 mm in length were excluded from the count. If artifacts were present on either side, only the branches on the unaffected side were counted.

### 2.5. Objective Analysis

The signal-to-noise ratio (SNR) and contrast-to-noise ratio (CNR) were measured for the LSA and PA regions on CTA images using kernels Hv40 and Hv72, with and without QIR, on the workstation. For the LSA region, the SNR and CNR were calculated by placing regions of interest (ROIs) in the proximal portions of the right and left MCA and in the ipsilateral white matter above the lateral ventricle in a 1-mm-thick coronal section. ROIs were consistently applied across all images with different kernels and QIRs to ensure uniform size and placement. For the PA region, ROIs were placed in the midsection of the basilar artery and pons, avoiding the vessels, in a 1-mm-thick sagittal section. One radiologist (T.N.) set these ROIs. The SNR and CNR were computed using the following formulas [17,28,29]:

$$\mathrm{SNR}={\mathrm{HU}}_{\mathrm{artery}}/\sqrt{{\mathrm{SD}}_{\mathrm{artery}}}$$

$$\mathrm{CNR}=({\mathrm{HU}}_{\mathrm{artery}}-{\mathrm{HU}}_{\mathrm{white}\;\mathrm{matter}})/\sqrt{{\mathrm{SD}}_{\mathrm{artery}}^{2}+{\mathrm{SD}}_{\mathrm{white}\;\mathrm{matter}}^{2}}$$

where HU represents the CT attenuation value and SD denotes the standard deviation within the ROI. The SNR and CNR values for the LSA region were calculated bilaterally and were averaged to obtain the final results.

### 2.6. Statistical Analysis

The scores from the visual inspection of the LSA and PA were compared across different CT reconstruction methods using the Friedman test, followed by post hoc pairwise comparisons. Statistical analysis was conducted with MedCalc Statistical Software version 22.021 (MedCalc Software Ltd., Ostend, Belgium; https://www.medcalc.org, accessed on 1 July 2024), with *p*-values < 0.05 considered statistically significant. Interobserver agreement between the two readers was evaluated using Gwet’s AC1 due to imbalances in score frequencies [30], with analysis performed using R statistical software (version 4.3.1; R Foundation, Vienna, Austria) and the irrCAC package, due to the lack of this analysis in MedCalc. Gwet’s AC1 was interpreted as follows: slight (0.01–0.20), fair (0.21–0.40), moderate (0.41–0.60), good (0.61–0.80), and almost perfect (0.81–1.0) [31]. Descriptive statistics are reported as median [range].

The SNR and CNR were compared among reconstruction methods for the LSA and PA regions, respectively, using the Friedman test with post hoc pairwise comparisons, owing to the dataset comprising normally and non-normally distributed data.

## 3. Results

### 3.1. Study Population

Initially, 31 patients who underwent UHR CTA on PCD-CT were identified. One patient was excluded due to a large sphenoid wing meningioma that compressed the cerebrum. Thus, 30 patients (8 males, 22 females; mean age, 59.1 years [range: 14–79]) were included in the analysis. The mean volume CT dose index was 28.6 mGy (range: 21.7–32.1 mGy). The CT examinations were performed for various reasons, including postaneurysmal clipping (n = 16), internal carotid artery aneurysm embolization with flow diverter placement (n = 3), aneurysm assessment for internal carotid artery (n = 3) and anterior communicating artery (n = 1), internal carotid artery stenosis (n = 2), preoperative assessment of trigeminal nerve schwannoma (n = 1), vertebral dissecting aneurysm embolization (n = 1), olfactory neuroblastoma (n = 1), and screening for ventricular calcification (n = 1) and lip arteriovenous malformation (n = 1). The counting of the LSA was not possible in one patient due to the presence of a coil. Quantitative analysis could not be completed for the right LSA region due to a clip (n = 1), for the bilateral LSA region due to a coil (n = 1), and for the left LSA region due to a flow diverter (n = 1).

### 3.2. Subjective Analysis

**Figure 2:**
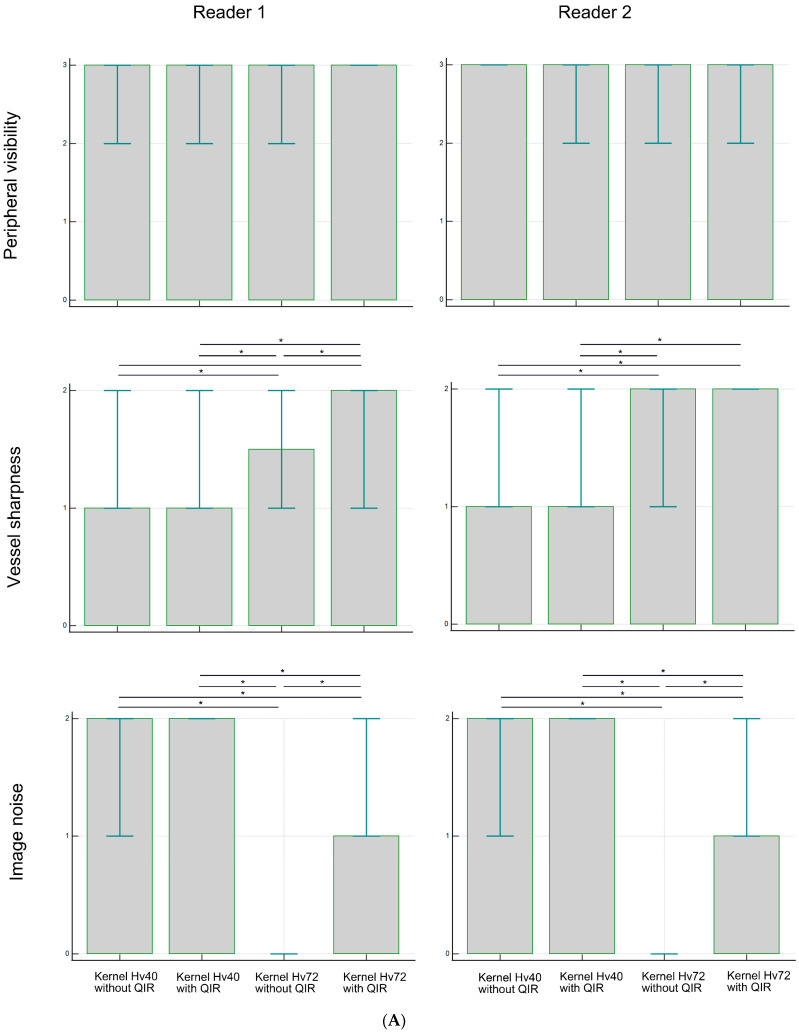

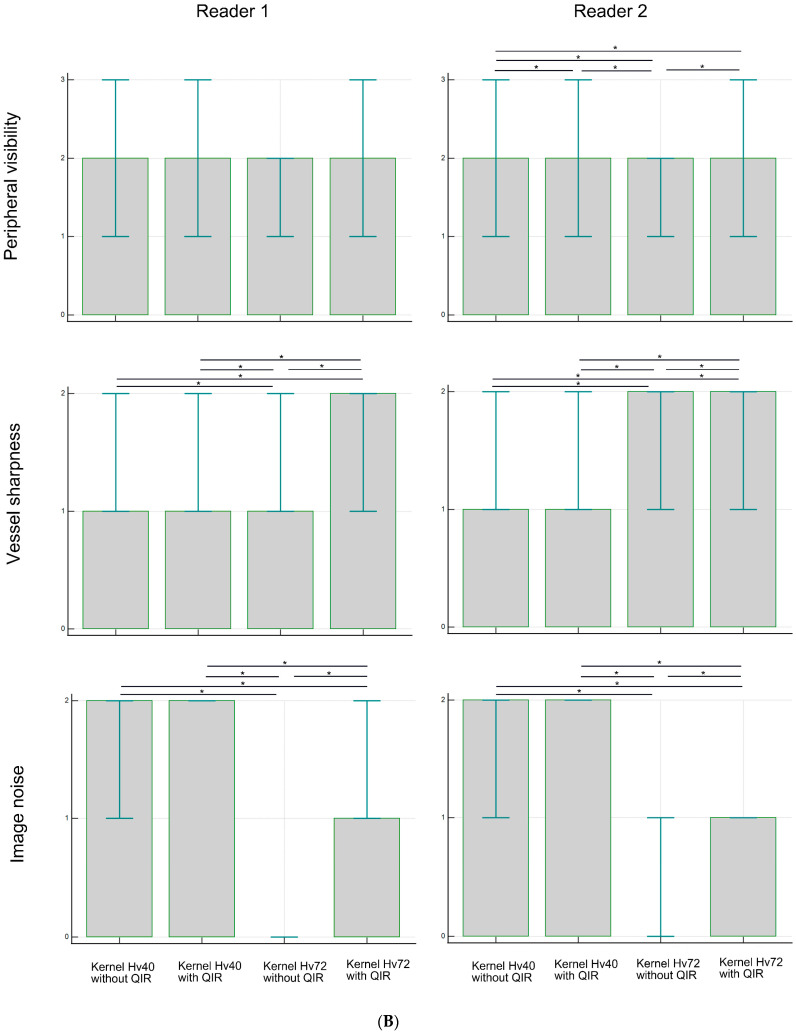
Subjective analysis scores for peripheral visibility, vessel sharpness, and image noise of the LSA (**A**) and PA (**B**) in UHR PCD-CTA, using kernel Hv40 with and without QIR and kernel Hv72 with and without QIR. The box indicates the median score, and the error bar shows the score range. * indicates significant differences with post hoc pairwise comparison.

**Figure 3:**
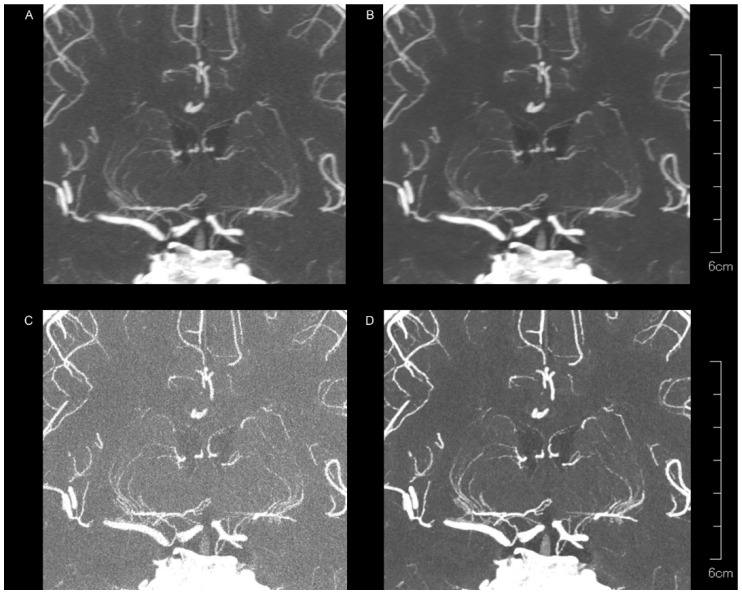
Representative UHR PCD-CTA images with coronal partial maximum intensity for a 68-year-old female, showing kernel Hv40 without QIR (**A**) and with QIR (**B**), as well as kernel Hv72 without QIR (**C**) and with QIR (**D**) in the LSA region. The LSAs are generally well visualized across all reconstruction methods, but vessel sharpness and image noise vary among methods.

Figure 2 and Table 1 present the subjective assessment results. Peripheral visibility scores for LSAs were consistently excellent across all reconstruction methods for both readers (all median scores were 3) and showed no significant differences among the methods (both *p* > 0.05). However, vessel sharpness scores for the LSA varied significantly among reconstruction methods (both *p* < 0.00001), with a better sharpness being observed with kernel Hv72 compared to kernel Hv40 (both *p* < 0.05). The image noise scores for the LSAs also differed significantly among reconstruction methods (both *p* < 0.00001), with higher noise scores being observed with kernel Hv40 compared to kernel Hv72 (both *p* < 0.05). The scores for kernel Hv72 without QIR, which were mostly 0, were significantly lower than those for other methods (both *p* < 0.05). Representative UHR PCD-CTA images of the LSA region are shown in Figure 3.

**Figure 4:**
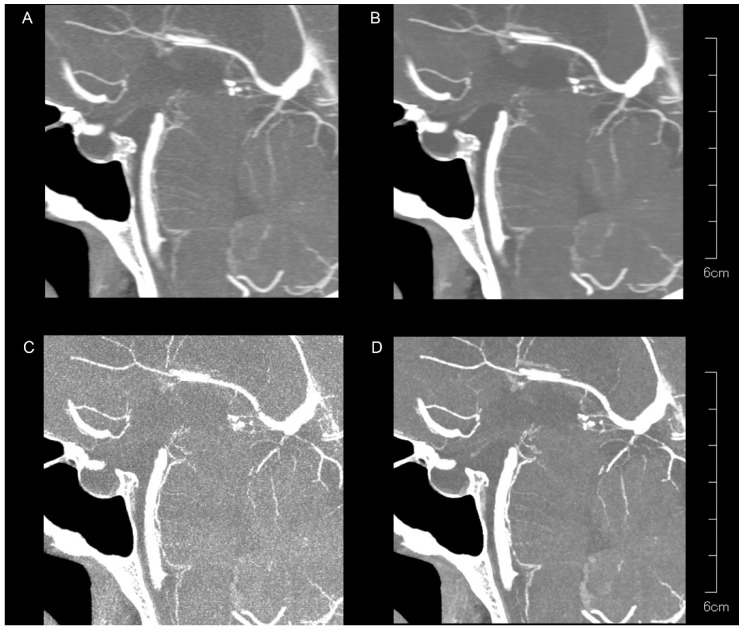
Representative UHR PCD-CTA images with sagittal partial maximum intensity projection for a 71-year-old female, showing kernel Hv40 without QIR (**A**) and with QIR (**B**), as well as kernel Hv72 without QIR (**C**) and with QIR (**D**) in the PA region. The PAs are less visible compared to the LSAs, with variations in vessel sharpness and image noise among the reconstruction methods.

For the PAs, the peripheral visibility scores were generally good across all reconstruction methods for both readers (all median scores were 2) but lower than those for the LSAs. The visibility scores for PAs did not differ among reconstruction methods for reader 1 (*p* = 0.257), but were higher for kernel Hv40 without QIR compared to other methods for reader 2 (*p* < 0.05). Vessel sharpness scores for PAs varied significantly among reconstruction methods for both readers (both *p* < 0.00001), with better sharpness being observed with kernel Hv72 with QIR compared to kernel Hv40 with and without QIR (both *p* < 0.05). For reader 1, kernel Hv72 with QIR also showed a better sharpness than kernel Hv72 without QIR (*p* < 0.05). The noise scores for PAs differed significantly among reconstruction methods for both readers (both *p* < 0.00001); kernel Hv40 with and without QIR (median score 2) had significantly higher noise scores than kernel Hv72 without QIR (median score 0) and kernel Hv72 with QIR (median score 1) in both readers (all *p* < 0.05). Representative UHR PCD-CTA images of the PA region are shown in Figure 4.

Interobserver agreement between the two readers was generally good or almost perfect (AC1 ≥ 0.61). Moderate agreement was noted for the vessel sharpness of the LSA on kernel Hv72 without QIR (AC1 = 0.600), the peripheral visibility of the PA on kernel Hv40 without QIR (AC1 = 0.600), the vessel sharpness of the PA on kernel Hv72 without QIR (AC1 = 0.567), and the vessel sharpness of the PA on kernel Hv72 with QIR (AC1 = 0.500).

**Figure 5:**
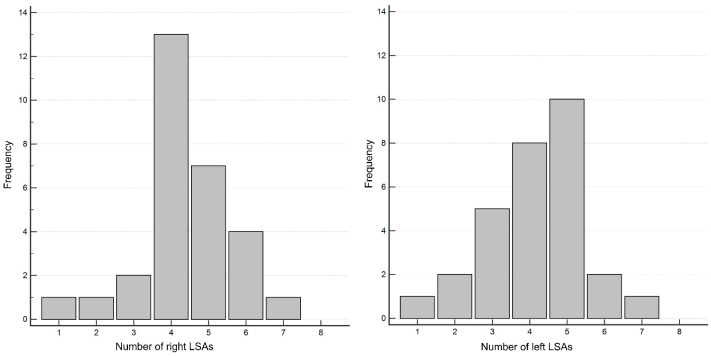
Distribution of the number of right (**A**) and left (**B**) LSAs.

The number of LSAs was counted in 29 patients. The average number of right LSAs was 4.4 (range: 1–7), and for left LSAs, it was 4.2 (range: 1–7) (Figure 5).

### 3.3. Objective Analysis

**Figure 6:**
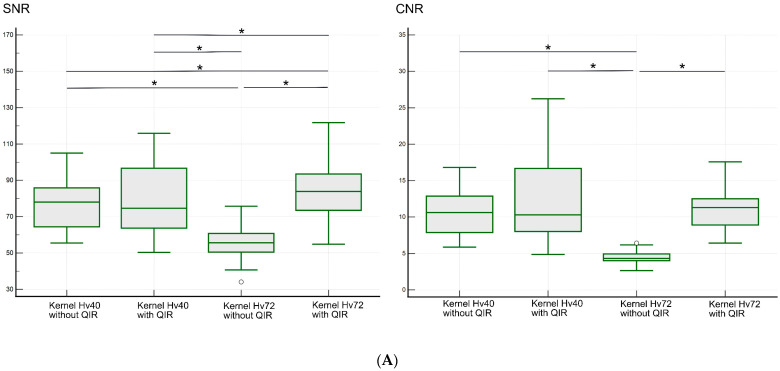

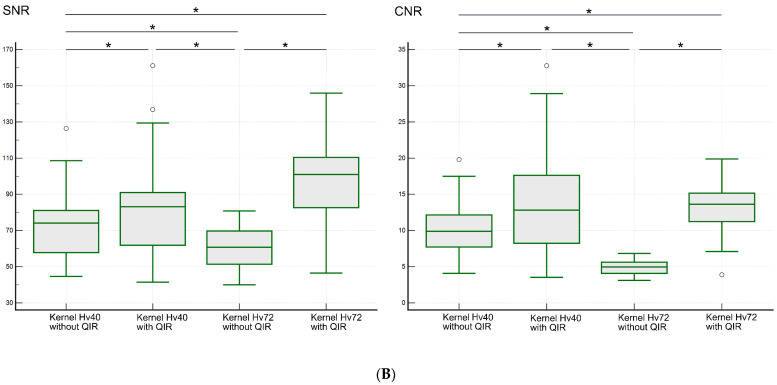
SNR and CNR values for the LSA (**A**) and PA (**B**) regions on UHR PCD-CTA using kernel Hv40 with and without QIR and kernel Hv72 with and without QIR. The data are shown as box-and-whisker plots, depicting the median, upper and lower quartiles, and the maximum and minimum values. * indicates significant differences with post hoc pairwise comparison. White circles indicate outliers.

The results of the objective analysis are shown in Figure 6 and Table 2. The median SNR (range) in the LSA region was 78.0 (55.5–105.0) for kernel Hv40 without QIR, 76.6 (50.3–115.9) for kernel Hv40 with QIR, 55.7 (34.1–75.8) for kernel Hv72 without QIR, and 85.4 (54.9–121.8) for kernel Hv72 with QIR. These values were significantly different (*p* < 0.00001). Post hoc analysis showed that the SNR was significantly higher for kernel Hv72 with QIR compared to other reconstructions, while the SNR for kernel Hv72 without QIR was significantly lower than for the other reconstructions (all *p* < 0.05). The median CNR (range) in the LSA region was 10.6 (5.9–16.8) for kernel Hv40 without QIR, 10.3 (4.9–26.2) for kernel Hv40 with QIR, 4.3 (2.6–6.4) for kernel Hv72 without QIR, and 11.3 (6.4–17.6) for kernel Hv72 with QIR. These values were significantly different (*p* < 0.00001). Post hoc analysis indicated that the CNRs for kernel Hv40 with and without QIR and or kernel Hv72 with QIR were similar (*p* > 0.05), whereas the CNR for kernel Hv72 without QIR was significantly lower than for the other reconstructions (*p* < 0.05).

The median SNR (range) in the PA region was 74.1 (44.6–126.4) for kernel Hv40 without QIR, 83.2 (41.5–161.1) for kernel Hv40 with QIR, 60.7 (40.0–80.8) for kernel Hv72 without QIR, and 101.0 (46.5–145.9) for kernel Hv72 with QIR. These values were significantly different (*p* < 0.00001). Post hoc analysis showed that the SNR was the highest for kernel Hv72 with QIR, followed by kernel Hv40 with QIR, kernel Hv40 without QIR, and kernel Hv72 without QIR (all *p* < 0.05). The median CNR (range) in the PA region was 9.9 (4.1–19.8) for kernel Hv40 without QIR, 12.8 (3.5–32.8) for kernel Hv40 with QIR, 5.0 (3.1–6.8) for kernel Hv72 without QIR, and 13.6 (3.9–19.9) for kernel Hv72 with QIR. Post hoc analysis revealed that the CNR was significantly higher for kernels Hv40 and Hv72 with QIR compared to those without QIR (*p* < 0.05), and the CNR for kernel Hv72 without QIR was significantly lower than for the other reconstruction methods (all *p* < 0.05).

## 4. Discussion

Our study demonstrated that LA peripheral visibility on UHR PCD-CTA was generally good and consistent across different kernels and QIR settings. This suggests that perforating arteries are effectively visualized regardless of the reconstruction method, likely due to the high-resolution capabilities of PCD-CT. However, vessel sharpness and image noise varied among the reconstruction methods. Kernel Hv72 provided sharper images of the perforators compared to kernel Hv40. This aligns with the fact that higher-numbered kernels are designed for sharper images. Sharpness is beneficial for visualizing fine arteries like the LSA, but it also increases image noise. Combining a sharp kernel with QIR can help in better depicting the LSA. Similar findings were reported by Tóth et al. [29], who observed that sharper kernels improved imaging quality for intracranial arteries and aneurysms. Our study confirms this trend for perforators, with higher-numbered kernels being advantageous for small vessels like the LSA.

The peripheral visibility of the PAs did not vary significantly among the different reconstruction methods, but the PAs were less visible compared to the LSAs. This difference can be attributed to several factors. First, the PAs are typically thinner than the LSAs (PA diameter ranges from 190 to 800 μm, while LSA diameter ranges from 100 to 2200 μm [32]). Second, there are fewer PAs compared to LSAs (mean number of PAs is 6.1, compared to 8.1 for LSAs [33]). Third, the PAs are situated closer to the skull than the LSAs, making them more susceptible to beam hardening artifacts and noise, which can impair visualization. Even with advanced PCD-CT technology, it remains challenging to clearly observe the distal PAs. The effects of reconstruction methods on vessel sharpness and image noise in the PA region followed the same pattern as observed for LSAs.

The distribution of perforators in the lenticular region primarily includes the medial striate artery (MSA) and the LSA. This study focused on the number of LSAs visible from the MCA using UHR PCD-CTA. The average number of right LSAs was 4.4 and the average number of left LSAs was 4.2. Previous anatomical studies reported an average of 8.1 LSAs (range: 1–21) [32,33], suggesting that not all LSAs may be visible with UHR PCD-CTA. On the other hand, a study using high-resolution energy integrating detector CT found an average number of 2.85 ± 0.83 LSAs [34]. This indicates that UHR PCD-CTA may provide a better visualization of LSAs compared to high-resolution energy integrating detector CT. However, the distribution of MSAs and LSAs can vary between individuals, and the number of perforators is inversely related to their diameter [32]. Therefore, more visible LSAs do not necessarily mean better visualization on CTA. Despite these variations, this study successfully identified several LSAs, including their distal portions, suggesting that UHR PCD-CTA can effectively evaluate small brain vessels. UHR PCD-CTA could serve as an alternative to angiography for diagnosing specific perforators and for exploring their relationship with certain lesions in the future.

The SNR and CNR of UHR PCD-CTA using kernel Hv72 without QIR were low in both the LSA and PA regions. Despite providing sharp vessel visualization, kernel Hv72 without QIR resulted in a low SNR and CNR, making it less suitable for CTA due to excessive noise. In contrast, CTA with kernel Hv72 with QIR generally showed a higher SNR compared to other methods. This improvement is likely due to the higher CT values in small vessels, especially near edges [29], and some noise reduction provided by QIR. The qualitative analysis results were consistent with the subjective analyses, though the direct analysis of perforators was not possible due to their thinness, which prevented ROI placement. Additionally, a sharpness assessment of the perforators could not be performed. Therefore, the SNR and CNR values in this study should be considered as reference points.

Combining visual evaluation with SNR and CNR data, kernel Hv72 with QIR appears to be the most effective for evaluating perforators compared to other reconstruction methods, despite slightly increased noise. The sharper kernel Hv72 with QIR offers a better definition and delineation of perforators. Previous reports suggest that kernels in the 40s are optimal for coronary arteries [35] and major neurovascular imaging [18,29], while sharper kernels are preferred for coronary plaque characterization and vessel lumen definition [17]. Tóth et al. [24] evaluated the intracranial major arteries on UHR PCD-CT with kernel 40s and QIR strength level 1. An excellent image quality was reportedly observed in most cases. However, the visibility of the perforators was not assessed. In this study, kernel Hv72 provided sharp images, which is beneficial for observing small vessels. Although sharper kernels introduce more noise, QIR helps reduce this noise [25], maintaining SNR and CNR at levels comparable to or better than kernel Hv40. While kernel Hv40 with QIR offers less sharpness but reduced noise and a similar CNR, it might be a suitable alternative, especially when evaluating both perforators and adjacent brain parenchyma. We believe that the results are very significant and suggest that PCD-CT can effectively visualize various perforators, aiding in detailed CTA assessments before surgery and in understanding vascular disorders.

The perforator visibility was investigated on magnetic resonance (MR) angiography, primarily on 5-T and 7-T MR units. The high-field MR units provide a high SNR and high-resolution images. Harteveld et al. [36] assessed LSAs and PAs on contrast-enhanced MR angiography on a 7-T MR unit and reported the mean (range) numbers of LSAs and PAs as 3.5 (1–8) and 5.0 (1–9), respectively. Kang et al. [37] investigated the visibility of PAs on 7T-MR angiography and reported that the number of PAs was 7.14 ± 2.79. Shi et al. [38] qualitatively evaluated the visibility of LSAs and PAs on MR angiography and reported that excellent or adequate visibility for diagnosis was obtained in all cases for LSAs and more than half of the cases for PAs on 5T and 7T units. Thus, the visibility of LSAs on UHR PCD-CTA is likely to be comparable to MR angiography on 5T or 7T. However, PA visibility may be lower on UHR PCD-CTA than MR angiography because the precise counting of PAs was difficult in our UHR PCD-CTA. The advantages of MR angiography include no irradiation, and no necessity of contrast agents, whereas the disadvantages include contraindication for metal devices, a relatively long scanning time, and a lower availability of such high-field MR units. Conversely, the CTA advantages include rapid scan time and no contraindication to patients with metallic devices; however, the disadvantages include irradiation, the necessity for a contrast agent, and bone-related artifacts, particularly in the posterior fossa. At present, there are no reports regarding the simultaneous comparison of the perforators between PCD-CTA and MR angiography. The CT and MR adaptations should be considered in the above-mentioned issues.

This study had several limitations. First, the included patients had arterial lesions or treatment devices, which might have impacted the visibility of the perforators. Additionally, the relatively older age of the subjects could have contributed to the reduction in the visualization of the perforators due to atherosclerosis. The small sample size might affect the generalizability of the findings. However, we assume that an adequate minimum number of cases was secured to detect the difference in the assessed scores. Second, contrast enhancement of the perforators may vary among patients due to differences in circulatory factors such as cardiac function, potentially leading to less enhancement in some individuals. Third, this study only analyzed kernels Hv40 and Hv72, without exploring intermediate kernels such as those in the 50s or 60s. In addition, the perforators themselves were only assessed qualitatively because of their thin structures. Experimental data, using phantom, may effectively investigate the relationship between the image quality and various reconstruction methods. Further research is needed to determine the optimal reconstruction parameters, including kernel types, QIR levels, and matrix sizes with larger sample sizes.

## 5. Conclusions

Intracranial perforators, especially the LSAs, were generally well depicted on UHR PCT-CTA. However, the PAs were less visible compared to the LSAs. While vessel sharpness and image noise varied among reconstruction methods, a sharp kernel combined with QIR appears to be the most effective for observing perforators.

## Figures and Tables

**Table 1:** Subjective analysis scores for peripheral visibility, vessel sharpness, and image noise of the LSA and PA in UHR PCD-CTA; data are presented as median (range).

**LSA**	**Kernel Hv40** **Without QIR**	**Kernel Hv40** **with QIR**	**Kernel Hv72** **Without QIR**	**Kernel Hv72** **with QIR**	***p***
Peripheral visibility					
Reader 1	3 (2–3)	3 (2–3)	3 (2–3)	3 (3–3)	0.293
Reader 2	3 (3–3)	3 (2–3)	3 (2–3)	3 (2–3)	0.109
Vessel sharpness					
Reader 1	1 (1–2)	1 (1–2)	1.5 (1–2)	2 (1–2)	<0.00001
Reader 2	1 (1–2)	1 (1–2)	2 (1–2)	2 (2–2)	<0.00001
Noise					
Reader 1	2 (1–2)	2 (2–2)	0 (0–0)	1 (1–1)	<0.00001
Reader 2	2 (1–2)	2 (2–2)	0 (0–0)	1 (1–2)	<0.00001
**PA**	**Kernel Hv40** **Without QIR**	**Kernel Hv40** **with QIR**	**Kernel Hv72** **Without QIR**	**Kernel Hv72** **with QIR**	***p***
Peripheral visibility					
Reader 1	2 (1–3)	2 (1–3)	2 (1–2)	2 (1–3)	0.257
Reader 2	2 (1–3)	2 (1–3)	2 (1–2)	2 (1–3)	0.00007
Vessel sharpness					
Reader 1	1 (1–2)	1 (1–2)	1 (1–2)	2 (1–2)	<0.00001
Reader 2	1 (1–2)	1 (1–2)	2 (1–2)	2 (1–2)	<0.00001
Noise					
Reader 1	2 (1–2)	2 (2–2)	0 (0–0)	1 (1–2)	<0.00001
Reader 2	2 (1–2)	2 (2–2)	0 (0–1)	1 (1–1)	<0.00001

**Table 2:** SNR and CNR values for the LSA and PA regions in UHR PCD-CTA; data are presented as median (range).

	Kernel Hv40 Without QIR	Kernel Hv40 with QIR	Kernel Hv72 Without QIR	Kernel Hv72 with QIR	*p*
LSA region					
SNR	78.0 (55.5–105.0)	76.6 (50.3–115.9)	55.7 (34.1–75.8)	85.4 (54.9–121.8)	<0.00001
CNR	10.6 (5.9–16.8)	10.3 (4.9–26.2)	4.3 (2.6–6.4)	11.3 (6.4–17.6)	<0.00001
PA region					
SNR	74.1 (44.6–126.4)	83.2 (41.5–161.1)	60.7 (40.0–80.8)	101.0 (46.5–145.9)	<0.00001
CNR	9.9 (4.1–19.8)	12.8 (3.5–32.8)	5.0 (3.1–6.8)	13.6 (3.9–19.9)	<0.00001

## Data Availability

The data supporting the findings of this study are available from the corresponding author upon reasonable request.
